# “Short term surgical complications after subthalamic deep brain stimulation for Parkinson’s disease: does old age matter?”

**DOI:** 10.1186/s12877-015-0112-2

**Published:** 2015-10-06

**Authors:** Vincenzo Levi, Giorgio Carrabba, Paolo Rampini, Marco Locatelli

**Affiliations:** Department of Surgery, Division of Neurosurgery, Fondazione IRCCS Caʼ Granda Ospedale Maggiore Policlinico, Milan, Italy

**Keywords:** Deep brain stimulation, Parkinson’s disease, Age, Complications

## Abstract

**Background:**

Patients aged 65 years and older are not traditionally considered optimal candidates for subthalamic deep brain stimulation (STN-DBS), mainly for their presumed increased incidence of surgical complications. The aim of this study was to assess STN-DBS surgery safety in relation to age.

**Methods:**

A total of 107 consecutive patients undergoing bilateral STN-DBS at our institution between 2002 and 2014 were retrospectively stratified according to age in two groups (Young group < 65 years old; Elderly group ≥ 65 years old;). Rate of short-term surgical complications (within 90 days) was reviewed and compared between the two groups.

**Results:**

Pre-operative baseline data were comparable between the two groups. The 90-days post-operative mortality rate was 0%. Overall incidence of complications related to surgery was 6,54%. In the Elderly group we observed 3 post-operative intra-cerebral haematomas (7,89%), 1 requiring urgent surgical evacuation. In the Young group we observed 2 post-operative asymptomatic intra-cerebral haematomas (2,89%) and 2 wound infections (2,89%), 1 requiring system removal. No others surgical complications were noticed in both groups.

**Conclusions:**

Chronological age ≥ 65 years old should not be considered alone as exclusion criteria to STN-DBS surgery.

## Background

Deep brain stimulation (DBS) can be considered one of the greatest revolutions occurred in the field of functional neurosurgery during the last 20 years [1]. Despite its therapeutic applications are expanding day by day, DBS main indication still remains the treatment of patients affected by advanced Parkinson’s Disease (PD). Among DBS standard targets, the subthalamic nucleus is considered the anatomical site of choice for the treatment of PD (STN-DBS) [2, 3].

Patients candidates for STN-DBS usually have a mean age at surgery of 58.6 years, a disease duration > 14 years and severe levodopa-induced motor complications [2–6]. Many neurosurgeons tend to avoid operating on patients aged 65 years and older because of their diminished “surgical tolerance”, which would be responsible for a raise of surgical complications [7–9].

It is well known, however, that PD incidence increases with time, and that mean age at diagnosis is 60 years [10, 11]. By 2030 patients affected by PD are expected to double [12]. Simultaneously, a quarter of the European population will be referred to as elderly (≥65 years old) [13, 14]. Considering these epidemiologic data, an increasing number of elderly patients will require STN-DBS for the treatment of PD in the next future. Guaranteeing an adequate evidence-based counselling by estimating the real surgical risks of this population will be paramount.

Although few prior studies suggested that the rate of surgical complications increases preferentially for older patients, scant literature has directly addressed the relationship between patient age and STN-DBS surgical adverse events in the PD population [15–17]. In addition, a recent study by DeLong et al. demonstrated a similar 90-days DBS surgical complication risk for elderly patients and younger counterparts, producing therefore contradictory findings [18].

Purpose of this study is to assess the potential negative role of age ≥ 65 years old on short-termsurgical complications in a specific population of patients undergoing STN-DBS for the treatment of PD.

## Methods

We retrospectively analysed surgical complications within 90 days in a cohort of 107 consecutive patients who underwent STN-DBS for the treatment of PD at our institution between 2002 and 2014. Selection criteria were the following: clinically diagnosed PD with severe motor fluctuations or dyskinesia not responsive to best medical therapies, a positive L-Dopa challenge test and a normal brain magnetic resonance imaging (MRI). Exclusion criteria were atypical parkinsonism, dementia or psychiatric impairment. For the purpose of this study, patients were stratified in two different subgroups according to age at time of surgery: patients younger than 65 years (Young group; *n* = 69) and patients aged 65 years and older (Elderly group; *n* = 38). The following permanent post-operative complications were reviewed: haemorrhagic and ischemic stroke, seizure, wound infection, hardware complication, pulmonary embolism and pneumonia. A Charlson comorbidity index ≥1 was used to assess the burden of risk factors in both groups [15].

Descriptive statistics were displayed as mean ± standard deviation for continuous data. Fisher’s exact test was used to compare qualitative data, with a *P* value set at 0.05. This was a large retrospective study without patient contact, required consent, or financial compensation.

### Surgical procedure

A stereotactic frame (Radionics CRW™ Stereotactic Frame, Integra, USA) was placed under local anaesthesia in the operating theatre after 24 hours medication withdrawal. A pre-operative stereotactic CT scan was usually fused with a cerebral MRI obtained the day before surgery. Volumetric T2-weighted sequences (slice thickness = 2 mm) and T1-weighted sequences with gadolinium were acquired in order to clearly localize subthalamic nucleus and avoid danger to vessels along the planned trajectory (Istereotaxy, Brainlab, Kapellenstrat, Germany).

A movement disorder neurologist performed intraoperative electrophysiological mapping and acute stimulation test in all cases. Microelectrode recordings were performed with 0,5 mm step checkpoints. Threshold for adverse events was routinely investigated.

Implantation of electrodes was always performed bilaterally in a single operation. The electrodes were then connected to a pulse generator (Kinetra o Activa Pc, Medtronic Inc, Minneapolis, Usa) 4 days later. Stimulation parameters and dopaminergic therapies were adapted postoperatively at 1, 6 and 12 months by a movement disorder neurologist.

## Results

Baseline characteristics among subgroups were found to be similar in terms of sex, disease duration, UPDRS III, UPDRS IV, Charlson comorbidity index and Levodopa equivalent dose (LED) [Table 1]. Mean age in the Elderly group was 68 ± 2,34 years ( range 65–75 years), compared to 57 ± 5,54 years of the younger counterpart (range, 42–64 years). The preoperative mean UPDRS III OFF in the Elderly group and the Young group was found to be 42 ± 12.34 and 44 ± 12,01 respectively (*p* = 0,7). Mean preoperative UPDRS IV dyskinesia subscore was slightly worse in the Young group (4 ± 2,10 versus 3 ± 1,50, *p* = 0,3). The preoperative UPDRS III assessed in ON state, instead, was higher for the Elderly group as expected, but the difference was not statistically significant (7 ± 5,59 versus 10 ± 5,57, *p* = 0,16). Mean disease duration of the entire cohort at the time of surgery was 13 ± 4,39 years (no differences between the two groups, *p* = 0,6).

**Table 1:** Demographic pre-operative charateristic of the two groups

	No	Sex (M/F)	Age	Disease duration	UPDRS III Off	UPDRS III On	UPDRS IV Dysk	UPDRS IV Fluct	LED	Charlson Index ≥1 (%)
Young	69	42/27	57 ± 5,5 (range 42–64)	13 ± 4,25	44 ± 12,01	7 ± 5,59	4 ± 2,06	4 ± 1,54	1415 ± 567,28	14 (20 %)
Elderly	38	25/13	68 ± 2,34 (range 65–75)	13 ± 4,68	42 ± 12,34	10 ± 5,57	3 ± 1,50	4 ± 1,41	1360 ± 289,07	12 (32 %)

The 90-days post-operative mortality rate was 0 %. Overall incidence of complications related to surgery was low (6,54 %) [Table 2]. In the Elderly group we observed 3 post-operative intra-cerebral haematomas, 1 requiring urgent surgical evacuation (7,89 % - 2 females and 1 male). In the Young group we observed 2 post-operative asymptomatic intra-cerebral haematomas (2,89 % - 2 males) and 2 wound infections, 1 requiring system removal (2,89 %,- 2 males). Transient post-operative confusion (<24 h) was noticed in 5 older male patients (13,15 %).

**Table 2:** Previous literature on elderly and DBS surgical complications

	Diagnosis	Type of procedure	Age criteria	No.	Hemorrhagic stroke	Infection	Hardware complication	Pneumonia or polmonary embulism	Seizure	Total	Follow-up	Conclusions
Voges et al. (2007)	PD Dystonia ET Others	DBS (nucleus not reported)	<60ys	528	1,9 %	N.r.	N.r.	0.5 %	N.r.	5.5 %	30-days	Age ≥ 60ys and PD risk factors for secondary surgical complications
			≥60ys	650	2,5 %	N.r.	N.r.	0,6 %	N.r.	7.7 %		
			Entire cohort	1,183	2.2 %	0,4 %	N.r.	0.6 %	0.4 %	6.8 %		
Derost et al. (2007)	PD	STN-DBS	<65ys	53	None	1.9 %	1.9 %	None	None	3.8 %	2 years	Age ≥65ys is not a surgical risk factor
			≥65ys	34	None	None	None	2.9 %	None	2.9 %		
			Entire cohort	87	None	1.9 %	1.9 %	2.9 %	None	3.4 %		
Rughani et al. (2013)	PD, Dystonia ET	DBS (nucleus non reported) Pallidotomy Thalamotomy	Entire cohort (correlation and logistic regression study)	5446	1.9 % risk for patients >70ys	N.r.	N.r.	N.r.	N.r.	3.5 % risk for patients >70ys	/	Age is correlated with in-hospital complications, but appears to serve as a surrogate for comorbidity. Diagnosis of PD carries an increased risk of in-hospital complications.
Shalash et al. (2014)	PD	STN-DBS	≤55ys	29	N.r.	N.r.	17.1 %	N.r.	N.r.	31.0 %	5 years	Age ≥65ys is not a surgical risk factor
			56-64ys	52	N.r.	N.r.	7.7 %	N.r.	N.r.	11.6 %		
			≥65ys	29	N.r.	N.r.	13.8 %	N.r.	N.r..	13.8 %		
			entire cohort	110	N.r.	N.r.	11.8 %	N.r.	N.r	17.2 %		
DeLong et al. (2015)	PD	DBS (nucleus non reported)	Entire cohort (logistic regression study per 5-ys increase)	1757	OR 0.82 (95 % C.I 0.63 1.07) *P* = 0.14	OR 1.04 (95 % C.I 0.87–1.24) *P* = 0.69	N.r.	OR 1.28 (95 % CI 0.99–1.64) *P* = 0,06	N.r	OR 1.10 (95 % C.I 0.96–1.25) *P* = 0.17	90-days	Age alone should not be a primary exclusion factor for determining candidacy to DBS.
Present study	PD	STN-DBS	<65ys	69	2,9 %	2,9 %	None	None	None	5.8 %	90-days	STN-DBS is a safe surgical procedure both in young and elderly patients
			≥65ys	38	7.9 %	None	None	None	None	7.9 %		
			Entire cohort	107	4.7 %	2.9 %	None	None	None	6.5 %		

Legend: PD (Parkinson’s disease); ET (Essential tremor); N.r. (not reported); OR (Odds ratio); CI (Confidence interval)

No other complications were observed in both groups. No statistically significant differences (*p* = 0,9) were noticed between the two groups in terms of post-operative complications.

## Discussion

In the western countries the term elderly is conventionally referred to as chronological age of 65 years and older [19]. Great improvements in health and social care have led to rapid growth of elderly population worldwide. By 2030, a quarter of the European population will be considered as elderly [14]. How this demographic change will impact boundaries of everyday clinical practice represents a new, stimulating field of research worldwide.

Elderly patients affected by PD are not traditionally considered optimal candidates for STN-DBS surgery [8, 9]. Despite evidence of equal motor fluctuation and dyskinesia reduction in elderly individuals, the mean age at STN-DBS surgery for the treatment of PD is 58.6 years [5, 15, 17]. One potential explanation could be that neurologists and neurosurgeons often consider concomitant disabling comorbidities of elderly subjects as STN-DBS relative contraindications [3, 7, 8]. In addition, there is evidence that elderly patients have higher rate of deterioration in axial symptoms such as freezing of gait and postural instability following surgery [15, 20, 21]. Finally, in case of major surgical complication, elderly patients may experience greater difficulty recovering and incresead rate of secondary adverse events, such as pneumonia or thromboembolism, because of their presumed decreased physiologic reserve [22, 23]. These considerations have led to exclude older patients from trials related to STN-DBS [4, 5]. Therefore, data about their “surgical tolerability” are limited to retrospective, nonrandomized studies [15–18, 20, 22, 24].

Although few prior studies suggested that the rate of surgical complications increases preferentially for older patients undergoing DBS for the treatment of different movement disorders, scant literature has directly addressed the relationship between patient age and surgical adverse events in a population of patients affected by PD undergoing STN-DBS [Table 2]. In a large multicenter study by Voges et al., the authors concluded that age ≥ 60 years and diagnosis of PD were risk factors associated to major surgical complications and secondary adverse events in a group of 1,183 patients treated for different movement disorders [22]. Similar findings have been reported by Rughani et al., who showed a negative prognostic role for old age on surgical mortality and complications in a study including a total of 5464 patients treated for PD, essential tremor and dystonia with deep brain stimulation or ablation procedures [25]. Interestingly, in the two studies the primary diagnosis of PD carried an increased likelihood of developing post-operative complications, in particular intracranial haemorrhage and pneumonia, if compared to other movement disorders [22, 25]. Whether the increased likelihood of intracranial haemorrhage may be direct consequence of the different surgical target selected (Pallidal Vs STN and thalamic DBS), the high prevalence of frailty syndrome in subject affected by PD could be one potential explanation for the high incidence of pneumonia [9, 23, 24, 26, 27]. One limitation of these studies was that they included different patient diagnosis (PD, essential tremor, dystonia), modality treatments (DBS, pallidotomy or thalamotomy), and targets (Stn, Gpi and thalamus). The potential negative impact of increased age on surgical complications in relation to the PD population undergoing specifically STN-DBS was not directly addressed. A recent large retrospective cohort study (*n* = 1757) by DeLong et al. produced contradictory findings on the argument, showing a similar 90-days complication risk for older parkinsonian patients and younger counterparts, suggesting that age alone should not be a criteria for selecting patients candidates for DBS [18]. It is to notice, however, that in this study the typology of neurostimulation is not reported (subthalamic, thalamic or pallidal).

Unlike previous published literature, the present study evaluates short-term surgical complication rate in relation to age in a specific group of patient undergoing STN-DBS. The 90-days mortality was 0 %. Overall incidence of permanent surgical adverse events was low (6,54 %). These data agree with prior estimates for DBS-related complications and confirm that STN-DBS, as a matter of fact, is a minimally invasive surgical technique. [3, 4] The cohort analysed in this study was characterized by comparable disabilities, motor impairments and risk factors at the baseline, differing only for age at surgery. STN was the brain target in all cases. Older patients had no higher incidence of surgical complications compared to the younger counterpart after STN-DBS. This finding is in line with other previous studies [15, 17, 20]. Therefore, from a strictly surgical point of view, at our institution chronological age of 65 years and older is not be anymore considered as a relative contraindication to STN-DBS surgery.

However, our data seem to suggest that surgical complications, especially intracranial haemorrhage, may be more severe in older patients. The only case in which an urgent craniotomy was required for post-operative intracranial haemorrhage, in fact, occurred in a 70-years subject with an unreported history of arterial hypertension. Conversely, the 2 post-operative intracranial haemorrhages observed in the Young group were discovered incidentally, being clinical asymptomatic. We also observed a higher rate of post-operative confusion in elderly subjects (13,15 % Vs 0 %). In all cases, the post-operative confusion was transient (<24 h) and therefore not considered as permanent surgical complication.

This study has some limitations due to its retrospective and nonrandomized design. Procedure bias, as healthier aged individuals being preferentially selected for surgical intervention, could have influenced our findings. Moreover, cognitive sequelae after STN-DBS has not been analysed because of the short-term period taken into account.

## Conclusions

In conclusion, our study shows that STN-DBS is a safe surgical procedure both in young and elderly patients. Overall rate of short-term surgical complications did not differ between patients aged < 65 years old and patients aged ≥ 65 years old. From a strictly surgical point of view, age alone should not considered as exclusion criteria to determine STN-DBS surgical candidacy. In the next future, multidisciplinary teams will be essential in order to assess the “biological age” of patients candidates to STN-DBS surgery and estimate their effective surgical risk.

## Abbrevations

DBS: Deep brain stimulation
STN-DBS: Subthalamic nucleus deep brain stimulation
PD: Parkinson’s disease
MRI: Magnetic resonance imaging
UPDRS: Unified Parkinson’s disease rating scale
LED: Levodopa equivalent dose
